# Local density and group size interacts with age and sex to determine direction and rate of social dispersal in a polygynous mammal

**DOI:** 10.1002/ece3.694

**Published:** 2013-08-01

**Authors:** Paula H Marjamäki, Adrienne L Contasti, Tim N Coulson, Philip D McLoughlin

**Affiliations:** 1 Department of Biological Sciences, Imperial CollegeSilwood Park, Ascot, Berkshire, SL5 7PY, UK; 2 Department of Biology, University of Saskatchewan112 Science Place, Saskatoon, SK, S7N 5E2, Canada

**Keywords:** Density dependence, feral horse, quorum sensing, Sable Island, scale, social dispersal

## Abstract

Movement away from an area or social group in response to increasing density (density-dependent dispersal) is known for most species; why it evolves is fundamental to our understanding of ecology and evolution. However, we have yet to fully appreciate how individuals of varying conditions (e.g., age and sex) might differently consider effects of density (quorum) when deciding to disperse or not, and scale dependence in their sense of quorum. We tracked movements of all individuals of a naturalized population of feral horses (*Equus ferus caballus*; Sable Island National Park Reserve, Nova Scotia, Canada) during a period of rapid population growth (*N* increased from 375 to 484 horses from 2008 to 2010). Permanent dispersal from breeding groups (bands) was positively density dependent for all age and sex categories with respect to local density (horses/km^2^, bounded by the 99th percentile of individual movements [8000 m]), but was negatively and positively density dependent for males and females, respectively, in relation to group (band) size. Dispersal was generally female biased, with the exception of foals which moved with their mothers (no sex effect), and for yearlings and subadults when band sizes were smaller than average, in which case males dispersed at higher rates than females. Dispersal distance was positively related to local density. We conclude that dispersal rate can be both positively and negatively density dependent for feral horses, contingent on the state of individuals and the scale at which quorum with respect to choosing to disperse or not is assessed. Scale effects and interactions of density-dependent and sex- and age-biased dispersal may have both ecological and evolutionary consequences through effects on resource and mate competition.

## Introduction

Density-dependent dispersal is known to almost always evolve (Travis et al. 1999), but how and why is still debated. A topic of recent research has been the direction of density-dependent dispersal, which can vary from positive to negative within taxa (Nowicki and Vrabec 2011) but also within populations (Kim et al. 2009; Pérez-González and Carranza 2009). Furthermore, condition dependency in dispersal rates and distances has garnered increased attention for a variety of species (e.g., insects [Hanski and Mononen 2011; ], lizards [Cote and Clobert 2010], birds, and mammals [review in Bowler and Benton 2005]), whereby the cost–benefit balance of dispersal hinges on environmental factors like density but also the internal state of an organism (body condition, sex, age, or discrete polymorphism in dispersal capacity). A general question arises: Can dispersal rate and distance be both positively and negatively density dependent within a population, contingent on the status of individuals? If so, what might this tell us about the selective pressures governing dispersal?

The difficulty with answering the questions above lies first in quantifying what Fellous et al. (2012) referred to as ‘quorum sensing’: Identifying the possible cues by which individuals perceive (or experience) population density in deciding whether to disperse, which may be similar to detecting Brown's (1988) ‘giving-up density’ in the context of foraging theory. This is a problem because we generally have little appreciation for how organisms might experience the selective pressures set upon them, including factors motivating dispersal. But we can expect that variability in selection pressure will scale in space and/or time (Chave 2013), and include conditions unique to individuals (Bélisle 2005; Travis et al. 2012). For example, the cues of quorum with respect to dispersal might vary according to individual perceptions of local exposure to conspecifics, which may include animals encountered directly or indirectly within an animal's home range or territory, but also directly within its own group if the species is social. Pérez-González and Carranza (2009) touch on this when they discuss effects of competition on individual decisions to disperse as a dichotomy between competition with conspecifics (demographical competition) versus competition with relatives (local or kin competition). In accordance, scale effects on dispersal have been recently noted by Roy et al. (2012) for black bears (*Ursus americanus*); and VanderWaal et al. (2009) observed that for African lions (*Panthera leo*) subadult females were most likely to disperse when intragroup competition was high (large pride size) and intergroup competition was low (fewer neighboring females in high-density habitat). Matthysen (2005) suggested that studies on density-dependent dispersal should consider multiple parameters of dispersal that capture variation in the dispersal process at different scales. We advocate explaining variation in dispersal probability or distance using individuals as the sampling unit (Larsen and Boutin 1994; VanderWaal et al. 2009; Travis et al. 2012), while taking into account each animal's state and unique experience of density, including local density and group size in how they might respond to the different cues of quorum.

**Figure 1:**
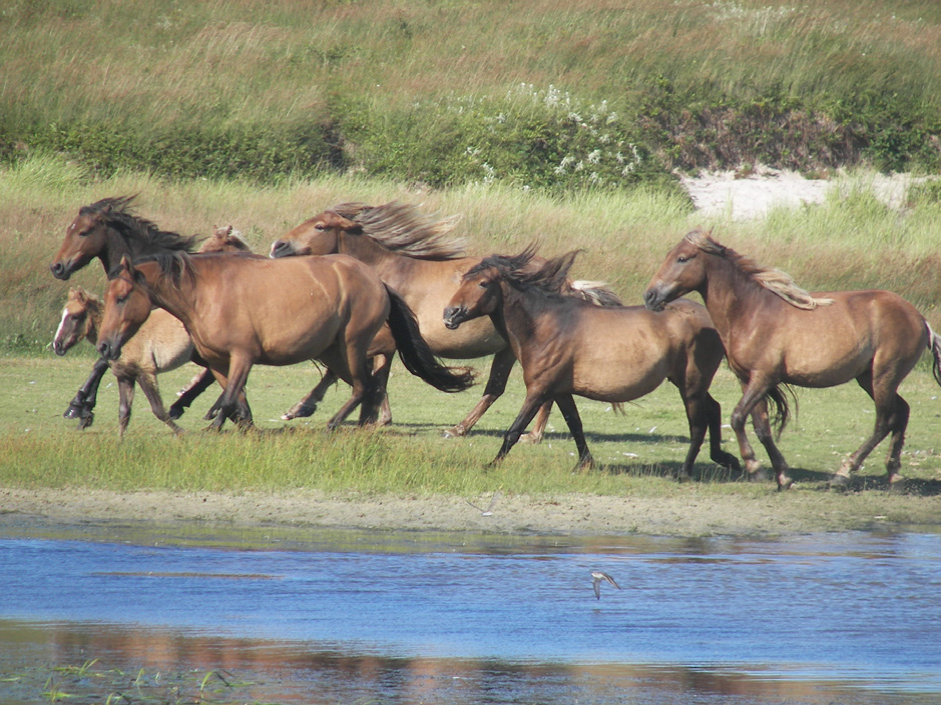
A band of Sable Island horses. Photo © Philip D. McLoughlin (2008).

**Figure 2:**
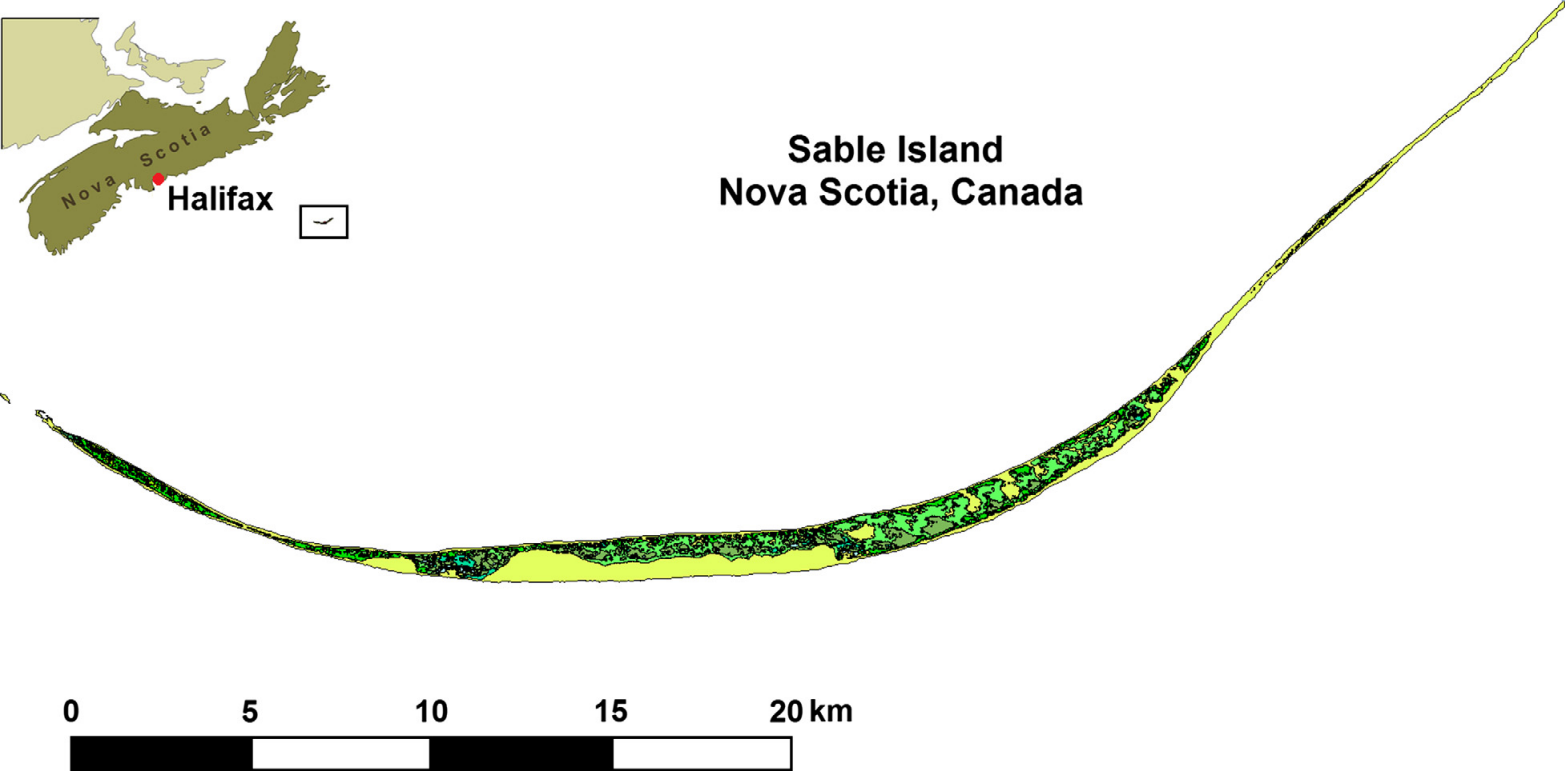
Sable Island National Park Reserve (43° 55′ N; 60° 00′ W), located approximately 275 km east–southeast of Halifax, Nova Scotia, Canada. The island is a crescent-shaped sand bar 49 km long and 1.25 km at its widest (vegetation in green).

Feral horses differ from other polygynous ungulates in that they form persistent, year-round breeding groups (bands) similar to that of some primates (Linklater et al. 1999). Total population density is known to increase dispersal distances for both males and females (Berger 1987). Little is known, however, of potential contrasting effects of increasing density versus band size on dispersal rates and distances, and how this might interact with sex and age of dispersers. Here, we examine how density influences rates and distances of social dispersal (permanent movements of individuals from one band to another, including both natal and secondary [breeding] dispersal) for an island population of feral horses (Sable Island, Nova Scotia, Canada, 2008–2010; Figs. 1, 2). For this we tracked the dispersal fates of every individual (classed by sex and four age categories) in the population (*N* = 375–484 from 2008 to 2010) while quantifying each animal's unique experience of: (1) the local density of breeding adults within 8000 m of its centroid of movement (radius containing 99% of within-summer movements of adult horses living in bands across all years of study); and (2) the number of horses in the band to which an individual belonged (group size). We selected these scales to capture the minimum and maximum range of individual experiences of density on the island: No horse used the entire island, and band size reflected density as experienced by individuals at the finest level of social organization.

We can expect density to affect individuals differently across scales as factors commonly thought to be important for dispersal may vary in this respect. For example, if the concern is exposure to competition for habitat resources (Morris and MacEachern 2010), density effects will include not only the number of individuals within a band but also competitors of other bands which share the same range (horses are not generally territorial, and possess overlapping home ranges [Berger 1986]). Here, we might expect horses to move into bands from areas of high local density to low and preferentially disperse to smaller bands, for example, if density reflects habitat quality and movements are in response to avoiding competition for resources. Competition for space and resources has been shown to be an important driver of habitat selection at both the coarse (dispersal) and fine (foraging) grain which amplifies as population density increases (i.e., density-dependent habitat selection; Rosenzweig 1981, 1991; Morris 2003; for Sable Island horses see van Beest et al. 2013). However, if the concern is inbreeding avoidance or kin and mate competition (Dobson 1982; Pérez-González and Carranza 2009; Cote and Clobert 2010; Clutton-Brock and Lukas 2012), composition of the band may be more important than the general local density within a horse's range. In all cases we anticipate sex differences in density-dependent dispersal given likely different costs for males and females to leave a band (Clutton-Brock and Lukas 2012). In horses, both sexes disperse and will move among bands (Berger 1986). However, whereas females will quickly disperse directly into another band, males often fail in this respect, dispersing into highly unstable ‘bachelor’ associations until they can attract females to establish a band, supplant an existing band stallion, or (re)join a band as a secondary ‘tag’ male (Berger 1986, 1987; Asa 1999; Linklater 2000). Because of this, natal dispersal may present different consequences for females versus males; and after natal dispersal, males, if they ever again are able to associate with a band, must also experience vastly different costs of secondary dispersal (note: here we do not consider interchange of males from one bachelor group to another or out of a bachelor group as ‘dispersal’ – bachelor group size and composition are highly ephemeral, and these interchanges [save movements into a band] are not likely to have consequences in terms of reproduction).

We hypothesize that for females, with high costs of gestation and lactation but relative ease in acquiring mates, resource competition will strongly affect dispersal patterns and so we predict effects of both density and band size on dispersal probabilities and distance. In addition, following Pérez-González and Carranza (2009), who proposed for red deer that sex-biased dispersal was related with male mate competition, we expect higher female (male) dispersal as male mate competition decreases (increases), as expected to occur at higher (lower) densities following Clutton-Brock et al. (1997). Furthermore, we hypothesize that males will be reluctant to move out of bands in response to increasing exposure to density because their concerns may be more in response to securing mating opportunities (Loe et al. 2009) or the high dispersal costs presented by male–male conflict (Berger 1987). Hence, we predict lower rates of dispersal in males relative to females in response to increasing local density (Kaseda et al. 1997), or possibly a positive direction in response to increasing band size (males will be more likely to stay within a band as it grows). For both sexes we expect juveniles to disperse at rates higher than adults as dispersal in mammals is expected to be highest immediately after individuals gain behavioral independence (Matthysen 2012).

## Materials and Methods

### Study area and sampling

**Figure 3:**
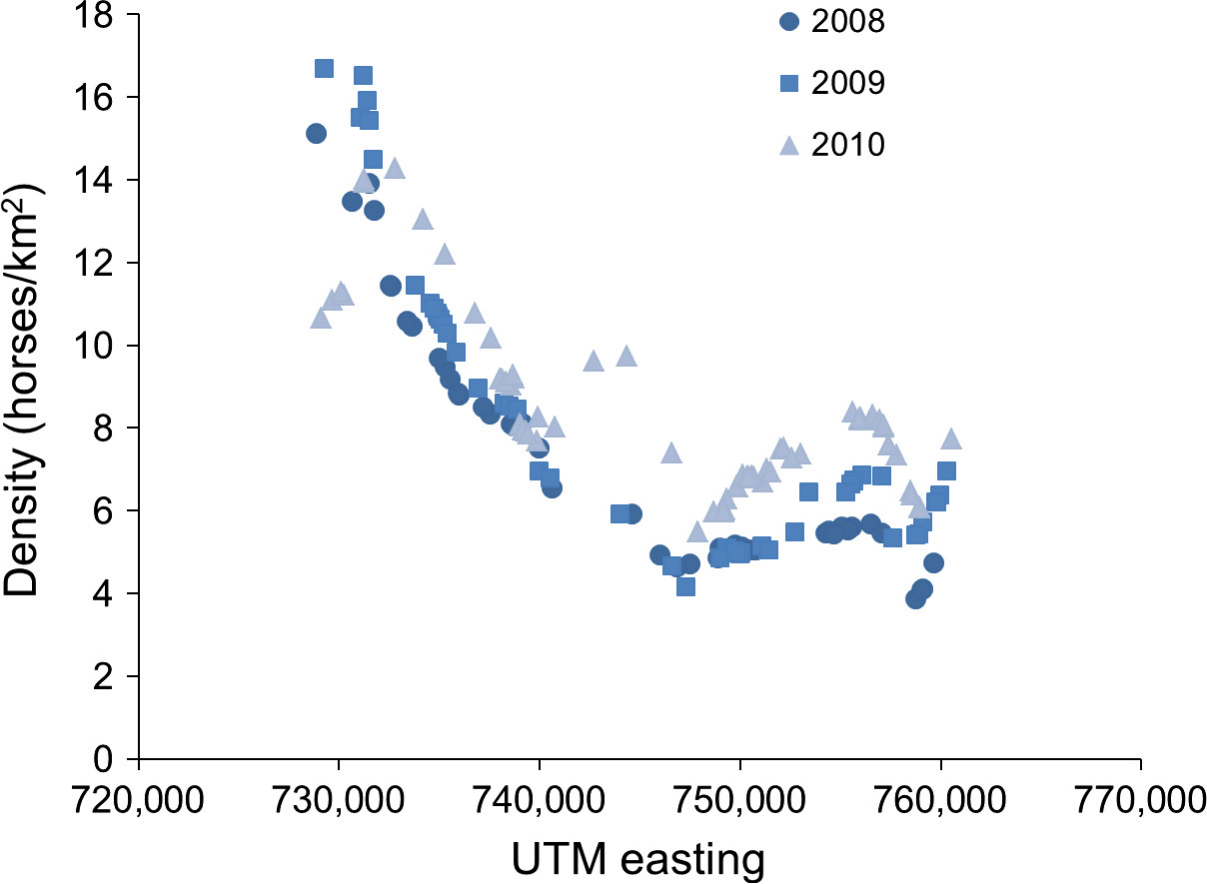
Variation in local density of adult horses (ages 4+) in bands (horses per km^2^ of vegetated habitat) versus a band's location (UTM easting [*x*]) on Sable Island, Canada (2008–2010). Each point represents the annual median *x*-*y* (UTM northing and easting) centroid of a band; local density is the number of adult horses in bands within 8000 m of each point.

Sable Island (43° 55′ N; 60° 00′ W) is a long (49 km) and narrow (1.25 at its widest in the center) sand bar located 275 km southeast of Halifax, Nova Scotia, Canada. The treeless island is characterized by wide beaches and grassy plains, hummocky heaths, and vegetated and bare sand dunes up to 30 m in height. Permanent water ponds are confined to the western and central parts of the island. In addition to greater access to freshwater in western Sable Island, important forage species such as sandwort (*Honckenya peploides*) and beach pea (*Lathyrus japonicus* var. *maritimus*) occur in the west at much higher densities. Contasti et al. (2012, 2013) and van Beest et al. (2013) describe the study area, including the west–east gradient that underlies variation in individual exposures to local density (Fig. 3).

Originally introduced sometime in the mid-1700s, Sable Island's feral horses have always been free ranging with minimal interference by humans (Contasti et al. 2012). We obtained our data, spanning years 2008–2010, as part of an ongoing individual-based study of the ecology and evolution of the horse population. We obtained direct observations of individual horses via systematic ground censuses of the entire horse population on Sable Island (observations from July to September). During each daily sampling effort (in one of eight sections of the island), we approached horses, which were ambivalent to humans, to within 5 m and recorded the location of an individual using a hand-held Global Positioning System (GPS), the horse's identity (verified using digital photographs), sex, reproductive status, and group membership. We classified field-aged horses as foals (age 0), yearlings (age 1), subadults (ages 2–3), and adults (ages 4+) based on appearance and known year of birth. If a horse was not observed during an entire season we assumed that it had died. We evaluated whether our censuses were adequate by comparing, for 2010 data, our summer counts of nonfoals with that obtained from high-resolution aerial photography in April 2010 (prior to births). This confirmed that our 2010 census accounted for >99% of the horses present in April. Our sampling was carried out under University of Saskatchewan Animal Care Protocol 20090032 following guidelines of the Canadian Council on Animal Care.

We only considered instances of permanent dispersal (i.e., temporary forays, separations, or excursions were excluded). Horses moving from one band through a series of bands in a season to the band that they finally settled in were counted as single moves. While we observed some permanent dispersal events directly during summer, the majority (86%) occurred between study seasons. We identified overwinter dispersal events by comparing band membership of individual horses between years: If a horse was recorded in a different band than in the previous year, it was considered ‘dispersed’. We considered only permanent emigration from bands (breeding groups) in our definition of dispersal because membership in adolescent and bachelor groups was highly unstable (dispersal to these groups was included). We considered density effects on both natal dispersal (emigration from a natal group to a breeding group) and secondary dispersal (subsequent emigration between breeding groups). Although our analysis incorporated age, we could not include dispersal type as a factor as we were unsure if natal dispersal had already occurred for yearlings or subadults in 2008 and 2009 (i.e., for horses that were born in 2007 or earlier [before our study commenced]). We excluded movements due to whole-band collapse, for example, because of a stallion's death (*n* = 2).

### Analysis

To investigate effects of local density and band size on the dispersal propensity of horses of different sex and age groups, we fitted a generalized linear model (GLM; with binomial errors) with dispersal (1 = dispersal, 0 = no dispersal) as the response variable. The maximal model included two factors (sex and age group) and two continuous variables (local density and band size) and all interactions. As no horse ranged across the entire length of the island, we measured the local density to which an individual was exposed as the number of adults in bands per km² of vegetated habitat within an 8000 m buffer (radius including 99% of all recorded, within-summer movements [8038 m]) of an individual's median centroid of movement, determined from a Geographical Information System (ArcGIS 9.3, ESRI, Redlands, CA, USA). We used the number of adults associated with bands in our measure of density as this would present a standard that excluded effects of transitory bachelor groups and better reflected the density of breeders encountered by individuals (males living outside of a band setting are highly unlikely to breed, e.g., Asa 1999; females were rarely encountered apart from bands). We used an ANCOVA to determine whether sex, age, band size, and local density affected an animal's dispersal distance. We obtained dispersal distances (m) by first calculating a median centroid of movement (median *x*-*y* coordinate) for each disperser for each band in which they were recorded (all members of the same band shared the centroid measure). We measured the distance between these subsequent locations for each disperser. We did not consider year effects as mean band size did not significantly differ among years of study, although average individual-based measures of local density increased from 7.2 to 8.5 horses/km^2^ during 2008–2010.

We generated minimal adequate models using the model simplification procedure described by Crawley (2007): First a maximal model was fitted (including all interactions), then we removed nonsignificant parameters, starting with highest-level interactions and nondiffering factor levels combined as appropriate. We retained nonsignificant main effects in the model if there were significant interactions. As both dispersers and nondispersers could belong to the same band with the same median centroid location, dispersal in the GLM was not an exclusive category as it usually applies to GLMs with binomial error (the distribution of dispersing individuals may be drawn from the same distribution of nondispersing individuals). Hence, to evaluate predictive capacity of our final GLM we adopted the approach of Boyce et al. (2002), which applies to this situation. We evaluated this model using *k*-fold cross-validation (test-to-training ratio of five data subsets), whereby we tested predictive capacity of partitioned models against withheld training data using the mean Spearman's rank correlation (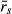) between training and test data, grouped within 10 bins (Boyce et al. 2002). We performed all analyses in R v. 2.15.1 (R Core Development Team 2012).

## Results

We observed 216 instances of dispersal by 168 individuals compared to 864 nondispersals (i.e., horses that did not leave their band despite opportunity) by 486 individuals (across years we relocated horses on 5307 occasions). We identified 60 events as natal dispersal. Dispersal was distributed across age categories (male, female) as: 36 foals (19, 17); 54 yearlings (24, 30); 75 subadults (28, 47); and 51 adults (6, 45).

**Figure 4:**
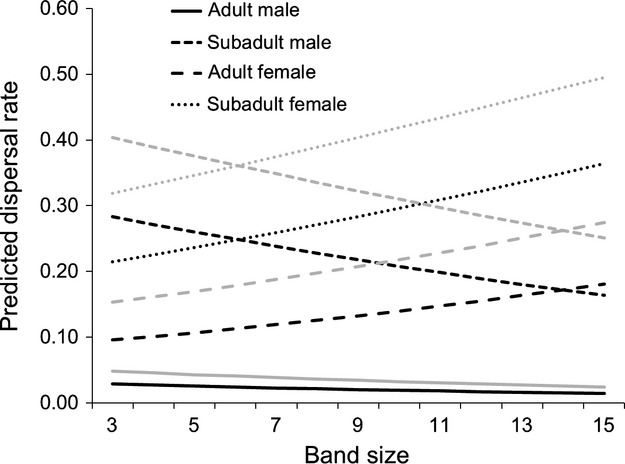
Interaction plots of dispersal probability versus band size for juvenile (yearling–subadult) and adult horses, Sable Island, Canada (2008–2010), at different local densities (low density in black and high density in gray). Density is measured as the number of adult horses in bands within 8000 m of an individual's centroid of movement (per km^2^ of vegetated habitat), presented as 1 SD below (low density: 5.11 horses/km^2^) and above (high density: 11.1/km^2^) the mean local density (8.1/km^2^).

Both band size (7.72 ± 2.90 horses [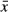 ± 1 SD], range 3–15) and a band member's exposure to local density (8.09 ± 2.98 horses/km^2^, range 3.86–16.69) were variable, but did not correlate (*R*^2^ = 0.008). Only density showed a trend in spatial heterogeneity, declining from west to east (Fig. 3). The final model for dispersal probability contained all main effects and two interaction terms (Table 1, Fig. 4). The yearling and subadult age classes could be combined (juveniles) without significantly reducing the model's explanatory power (χ^2^ = 5.27, df = 8, *P* = 0.73). The minimal adequate model indicated that local density had a highly significant, positive effect on whether a horse dispersed or not (all age and sex categories, regardless of band size; Table 1, Fig. 4), and removing it from the model significantly reduced the fit (χ^2^ = 13.13, df = 1, *P* < 0.001). Sex and age also had a significant effect on dispersal probability (χ^2^ =29.68, df = 12, *P* = 0.003; and χ^2^ = 84.80, df = 16, *P* < 0.001, respectively): Females of an age category generally had higher dispersal rates (proportion dispersing per year) than males (0.23 and 0.16, respectively). Juveniles had the highest dispersal rates (0.32), followed by foals (0.18) and adults (0.11). Model comparisons showed the interaction between sex and age to be significant (χ^2^ = 13.52, df = 2, *P* = 0.001, Fig. 4). Female juveniles and adults dispersed at rates greater than respective males (females: juvenile 0.34 and adult 0.16; males: juvenile 0.28, adult 0.03). Movements of foals from one band to another were facilitated in 34/36 instances by that of their mothers: Female and male foals moved at rates of 0.18 and 0.17, respectively, which were not significantly different. Overall, annual dispersal rate in the population was 0.20. The minimal adequate model also showed a significant difference between the sexes on effect of band size (band of origin) on likelihood of dispersal (χ^2^ = 4.13, df = 1, *P* = 0.04; Table 1, Fig. 4): Males showed a negative propensity to disperse relative to females as band size increased, but the opposite was true for females. Furthermore, at low band sizes juvenile males were expected to disperse at rates greater than females, although this reversed as band sizes increased (Fig. 4). All other interaction terms were nonsignificant (*P* > 0.10). Predictive capacity of the final model (Table 1) using withheld testing data was good (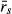 = 0.828).

**Table 1:** Minimal adequate model coefficients and significance of coefficients for the generalized linear model explaining dispersal probability for foals (age juvenile = 0, age adult = 0); yearlings and subadults (age juvenile = 1, age adult = 0); adults (age juvenile = 0, age adult = 1); and by sex (Sex M = 1, F = 0) for feral horses on Sable Island, Canada (2008–2010). Null deviance was 1080.97 on 1079 df; residual deviance was 977.07 on 1069 df

	Estimate	SE	*z*	*P*
Intercept	−2.829	0.447	−6.333	<0.0001
Sex M	0.954	0.598	1.594	0.111
Age Juvenile	0.878	0.305	2.877	0.004
Age Adult	−0.073	0.316	−0.232	0.817
Band Size	0.061	0.035	1.778	0.075
Density	0.093	0.025	3.649	0.0003
Sex M × Age Juvenile	−0.226	0.430	−0.526	0.599
Sex M × Age Adult	−1.866	0.585	−3.193	0.0014
Sex M × Band Size	−0.120	0.059	−2.040	0.041

The size of bands that dispersing individuals moved into was significantly smaller (6.63 ± 0.21 [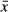 ± 1 SE]) than that of the band they left 8.31 ± 0.21 (band-to-band movements only; paired *t*-test, *t* = 6.32, df = 176, *P* < 0.0001). Dispersal distance ranged from 108 to 18023 m (3433 ± 3283 m [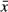 ± 1 SD]). Our final linear model included only the local density at origin of a disperser as a positive predictor of dispersal distance (*y* = 1357.6 + 235.4[density], *P* = 0.0006) although *R*^2^ was low (0.054).

## Discussion

Horses of all ages and both sexes increased rates of social dispersal in response to increasing local density. Age and sex also influenced the direction and slope of the relationship between dispersal rate and band size, which was negative for males and positive for females. Furthermore, slope was steeper for juveniles (yearlings and subadults) compared to adults. The interactions we observed hint at the varying selective pressures (which define quorum) that individuals might respond to when deciding to disperse.

The clear difference between male and female dispersal rates with respect to increasing band size may reflect the dynamics of mate competition within bands. Most bands (83.3%) contained only one stallion (compared to two [14.8%] and three [1.8%] adult males). Hence, with increasing band size, opportunities for males to gain access to mates likely increased, whereas for females, the opposite was true. Traditional mate competition theory generally applies to feral horses, with the higher investing sex (females) being limiting and thus competed for by males (Trivers 1972). Where males may have higher breeding and/or maintenance costs relative to females, they are expected to enter or remain in the breeding population at a lower rate than females as density increases. Consequently, the operational sex ratio at higher densities will be female biased, lessening competition for mates, and allowing remaining males to obtain larger harems (Clutton-Brock et al. 1997). This is expected for several polygynous ungulates, including red deer (Bonenfant et al. 2004), feral horses (Kaseda and Khalil 1996), and reindeer, *Rangifer tarandus* (Røed et al. 2002). The reluctance of males to leave bands should thus strengthen with increasing band size, but at the same time the attraction to stay in a band should lessen for females, and choosiness should increase because of high female costs of breeding (Kokko and Monaghan 2001). The crossing interactions for sex observed in this study with respect to dispersal rate versus band size (Fig. 3) provide empirical support for this hypothesis. In addition, the data support the findings of Pérez-González and Carranza (2009) who proposed for red deer that female dispersal (distance vs. genetic relatedness) was an inverse function of male mate competition, assuming mate competition is lower when harems are larger. The role of sex ratio on these decisions requires further research, however, as local density and band size were not correlated.

Greenwood (1980) suggested that philopatry will favor the evolution of cooperative traits between members of the sedentary sex, but that disruptive acts will be a feature of dispersers. It is possible that these traits may be a function of average band size in horses. Juvenile males dispersed at greater rates than females at less than average band sizes. Smaller bands, with higher potential for mate competition among males, may predispose individuals to have higher testosterone levels, leading to higher dispersal rates of young males, a hypothesis first suggested by Pérez-González and Carranza (2009). If true, this would support Greenwood (1980) in a proximal sense, and is worthy of pursuit given our results. Stallions may also be less likely to tolerate juvenile males in bands of smaller size if these bands are less likely to contain a band stallion's male offspring, as competitive band takeovers often lead to band fission (i.e., a large band is rarely kept intact during a takeover). Large bands may thus be led by a stallion that is more likely to consider juveniles as his own offspring.

Higher rates of female dispersal as band size increases are also consistent with strategies that reduce average kinship between group members (due to dominance and tenure of a stallion). This may explain why horses do not cooperatively breed (Linklater et al. 1999) and rarely show allomaternal behavior despite living in relatively persistent groups. Further testing, however, is required to assess how kinship might vary with band size by sex, but also by local density which is tied to habitat on the island. Contasti et al. (2012) documented higher densities of females (all ages) in western Sable Island (25.1 females/km^2^) compared to central (9.0 females/km^2^) and east areas (12.8 females/km^2^). We also show this trend using individual-specific local density estimates (Fig. 3). These trends matched Lucas et al.'s (2009) observation of higher levels of inbreeding (*F*_IS_ = 0.113) in western Sable Island compared to the east (*F*_IS_ = −0.008), suggesting a role for kin competition in driving social dispersal in horses which requires further research. However, lack of a relationship between band size and local density suggests that role of kinship in social dispersal may differ depending on scale of observation.

Whereas competition within bands may be explained by mate competition, positive density dependence for all conditions of sex or age in the context of local density suggests that all horses reacted to increasing competition for resources. Resource competition is a strong predictor of the movements and dispersal propensity of animals – especially female mammals with high costs of gestation and lactation. Density-dependent habitat selection is a well-known phenomenon with a long history of theoretical and empirical support (Rosenzweig 1981; Morris and MacEachern 2010). In habitat-selecting species, individuals are expected to be attracted first to the highest-quality habitat in a population's range. Consequently, high-quality habitat reaches higher density faster than low-quality habitat. In response to increased competition for resources, it may then become beneficial for some individuals to disperse to areas that are farther from local carrying capacity. Contasti et al. (2012, 2013) showed that during the 2008–2010 phase of population growth on Sable Island, females in the higher density west were producing and exporting more recruits than anywhere else on the island, with emigration from the west directed into central and eastern areas where there was less available fresh water and availability of high-quality forage. Population growth (λ) was heterogeneous between regional habitat zones, being lower (due to emigration) in the high-density habitat in the west (λ = 1.09) compared to central (λ = 1.16) and east (λ = 1.17) areas (both sexes, Contasti et al. 2012). Density-dependent habitat selection was confirmed in this population by van Beest et al. (2013).

If horses on Sable Island disperse in response to density-related effects of resource competition, we would expect density to have a negative effect on the survival and/or reproduction of individuals. For a large ungulate like the horse, offspring and juvenile survival are expected to the most sensitive parameters to density effects (Gaillard et al. 1998). Scorolli and Lopez Cazorla (2010) observed that density affected fecundity and yearling female survival in Argentinian feral horses, and the influence of density on offspring survival has been noted in other ungulates (e.g., Coulson et al. 1997). In support that movements of females were related to resource competition, Contasti et al. (2012) reported that foal survival was lower in the higher density west (0.823) compared to central (0.938) and east (0.954) Sable Island. Males as well as females will move in response to competition for food or water, but for a polygynous mammal like the horse we might expect males (without gestation and lactation costs) to not follow density gradients as strongly as females, as suggested by Figure 4.

It follows that the pressures for dispersal varied among different age groups (Clobert et al. 2009). Young individuals often tend to be subordinate and thus likely sensitive to competitive interactions (Bowler and Benton 2005) and kin competition. The pressure to disperse before (or at) sexual maturity in order to avoid inbreeding may be a key driving force of juvenile dispersal, as has been suggested for several mammalian species (Matthysen 2012). Inbreeding avoidance has been suggested to be the main cause of natal dispersal in feral horses, at least for females. Monard et al. (1996) showed that female natal dispersal in Camargue feral horses was closely related to sexual maturity, but no evidence was found to suggest dispersal to escape intraspecific competition. Similarities were found for subadult females on Assateague Island (Maryland, USA; Rutberg and Keiper 1993), and female dispersal in the Kaimanawa Range (New Zealand; Linklater and Cameron 2009) correlated with sexual receptivity.

We observed 3433 m as the mean dispersal distance, suggesting dispersing horses tended to move out of their original band's home range (radius of within-summer movements of horses relative to their centroids averaged 1290 ± 1649 m ([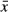 ± 1 SD]). Dispersal distance is expected to vary according to the ultimate cause and costs of dispersal (Baker and Rao 2004; Bonte et al. 2012), and density effects on dispersal distance are not unusual (Matthysen 2005). Berger (1987) reported positive density dependence on dispersal distance for feral horses, and we also detected a significant response here; however, explanatory power of our relationship was low. But if we were to consider movements across a much larger scale of density using a metapopulation framework, for example, adopting Contasti et al.'s (2012) west, central, and east divisions of Sable Island, movements among these spatially distant zones are significantly linked to density (Contasti et al. 2012, 2013; van Beest et al. 2013). Chave (2013) points out that ecological dynamics are always stochastic at small scales, but variability is conditional on the scale of description. A larger scale with less variability among individuals appears to be needed to observe trends in dispersal distance, compared to the question of whether a horse should abandon a local area of familiarity or group.

The broad-scale treatments of horse movements on Sable Island by Contasti et al. (2012, 2013) and van Beest et al. (2013) are more in line with the discrete nature in which density-dependent dispersal has thus far been addressed by most researchers (e.g., Amarasekare 2004a,b; Clobert et al. 2009). Our approach in this study was to adopt an individual-based perspective, which may risk some confusion with respect to terminology (hence our reference to social dispersal and dispersal distance, as opposed to immigration or emigration). However, because selection acts on individuals and not subpopulations, we believe the methods we present here are more in line with the current state at which ecology and evolution are being married, for example, to understand eco-evolutionary dynamics (Schoener 2011).

We demonstrate that dispersal and the direction of density dependence is not fixed but is both scale and condition dependent, varying according to the age and sex of an individual but also how they might perceive density effects (considering local density and group size). The overall positive density-dependent response is likely to influence the population dynamics of Sable Island. Scale effects and interactions of density-dependent and age- and sex-biased dispersal may have both ecological and evolutionary consequences through effects on resource and mate competition.
